# Development of proteoglycan-induced arthritis depends on T cell-supported autoantibody production, but does not involve significant influx of T cells into the joints

**DOI:** 10.1186/ar2954

**Published:** 2010-03-18

**Authors:** Adrienn Angyal, Colt Egelston, Tamás Kobezda, Katalin Olasz, Anna László, Tibor T Glant, Katalin Mikecz

**Affiliations:** 1 Section of Molecular Medicine, Department of Orthopedic Surgery, Rush University Medical Center, 1735 West Harrison Street, Chicago, IL 60612, USA; 2 Department of Immunology/Microbiology, Rush University Medical Center, 1735 West Harrison Street, Chicago, IL 60612, USA

## Abstract

**Introduction:**

Inflammatory joint destruction in rheumatoid arthritis (RA) may be triggered by autoantibodies, the production of which is supported by autoreactive T cells. Studies on RA and animal models of the disease suggest that T cells recruited in the joints can locally initiate or propagate arthritis. Herein, we investigated the role of joint-homing versus lymphoid organ-homing T cells in the development of proteoglycan-induced arthritis (PGIA), an autoimmune model of RA.

**Methods:**

To identify T cells migrating to the joints before and during development of autoimmune arthritis, we transferred fluorescence-labeled T cells, along with antigen-presenting cells, from BALB/c mice with PGIA to naïve syngeneic severe combined immunodeficient (SCID) mice. We then monitored the recruitment of donor T cells in the ankle joints and joint-draining lymph nodes of the recipients using in vivo two-photon microscopy and ex vivo detection methods. To limit T-cell access to the joints, we selectively depleted T cells in the blood circulation by treatment with FTY720, an inhibitor of lymphocyte egress from lymphoid organs. Reduction of T cell presence in both lymphoid organs and blood was achieved by injection of donor cells from which T cells were removed prior to transfer. T and B cells were quantitated by flow cytometry, and antigen (PG)-specific responses were assessed by cell proliferation and serum antibody assays.

**Results:**

Despite development of adoptively transferred arthritis in the recipient SCID mice, we found very few donor T cells in their joints after cell transfer. Treatment of recipient mice with FTY720 left the T-cell pool in the lymphoid organs intact, but reduced T cells in both peripheral blood and joints. However, FTY720 treatment failed to inhibit PGIA development. In contrast, arthritis was not seen in recipient mice after transfer of T cell-depleted cells from arthritic donors, and serum autoantibodies to PG were not detected in this group of mice.

**Conclusions:**

Our results suggest that antigen-specific T cells, which home to lymphoid organs and provide help to B cells for systemic autoantibody production, play a greater role in the development and progression of autoimmune arthritis than the small population of T cells that migrate to the joints.

## Introduction

Rheumatoid arthritis (RA) is a systemic autoimmune disease involving mainly the peripheral synovial joints and causing chronic inflammation and profound tissue destruction in affected patients [1]. The autoimmune character of RA is best supported by the presence of circulating autoantibodies (autoAbs) against immunoglobulins (rheumatoid factor), citrullinated proteins, and other endogenous proteins [2,3], which may become detectable in serum years before the development of joint symptoms [4]. The systemic production of autoAbs indicates that autoreactive T cells that provide help to B cells for Ab secretion are located in the secondary lymphoid organs and therefore are indirectly involved in disease pathogenesis. However, studies suggest that T cells recruited in the joints of RA patients may be directly involved in the initiation and propagation of arthritis [3,5].

Induced autoimmune animal models of RA, including collagen-induced arthritis (CIA), glucose-6-phosphate isomerase (G6PI)-induced arthritis, and proteoglycan (PG)-induced arthritis (PGIA), are known to involve major histocompatibility complex (MHC) II-restricted antigen (Ag) presentation and generation of T cells and autoAbs that cross-react with self-(auto)Ags such as mouse type II collagen (CII), G6PI, and mouse PG (mPG) [6-10]. Both CIA and PGIA can be adoptively transferred to syngeneic immunocompromised mice by lymphocytes isolated from arthritic donors [11-13]. Despite the autoimmune pathogenesis and development of robust and sustained inflammation of multiple joints in CIA or PGIA, the proportion of T cells present in the synovial fluid of these joints has been reported to be small [14,15]. However, with regard to autoimmune diseases, the consensus is that upon entry into the joints from the bloodstream, 'armed' effector T cells can provide cytokine/chemokine stimuli to surrounding cells and act in concert with these cells to trigger and maintain a local inflammatory process [16,17].

To address the importance of joint-homing versus lymphoid organ-homing T cells in PGIA, we took two experimental approaches. First, using *in vivo *two-photon microscopy (TPM), we monitored the migration of fluorescence-labeled T cells into the ankle joints and joint-draining lymph nodes (JDLNs) of syngeneic severe combined immunodeficient (SCID) mice during the course of the adoptive transfer of PGIA. TPM has been successfully used to visualize the rapid influx of T cells into the central nervous system upon induction of experimental allergic encephalomyelitis (EAE) [18,19], an animal model of multiple sclerosis (MS). However, in the adoptively transferred model of PGIA, we could hardly detect any T cells within the synovial tissue of the joints of SCID mice by TPM imaging either before or after arthritis development. The lack of synovial T cells was confirmed by immunohistochemistry (IHC) performed on tissue sections of the same joints, but a small population of T cells could be identified in synovial fluid samples of inflamed joints by flow cytometry. Second, to determine whether the availability of T cells in the circulation affects their migration into the joints and arthritis development, we used FTY720, a drug known to 'deplete' T cells in peripheral blood by inhibiting their exit from lymphoid organs [20-22]. FTY720, a sphingosine 1-phosphate (S1P) receptor modulator [20], has been found to be effective in preventing or suppressing EAE in rodents [23] and shows a strong therapeutic potential in MS [24]. In adoptively transferred PGIA, we found that FTY720 treatment of SCID mice, transferred with arthritic donor lymphocytes, effectively reduced T-cell presence in both the circulation and synovial fluid but did not inhibit or delay the transfer of arthritis. In contrast, SCID mice receiving T cell-depleted cells from the same arthritic donors failed to develop arthritis, suggesting a strict requirement for substantial T-cell presence for disease induction at locations other than the peripheral joints.

## Materials and methods

### Mice, immunization, and assessment of arthritis

Adult female BALB/c mice and female SCID mice (on the BALB/c genetic background) were purchased from the National Cancer Institute (Frederick, MD, USA). Enhanced green fluorescent protein-lysozyme M knock-in (EGFP-LysM KI) mice (on the C57Bl/6 background) [25] were obtained from the University of Missouri Mutant Mouse Regional Resource Center (Columbia, MO, USA) and were back-crossed to BALB/c for 10 generations. Mice were immunized intraperitoneally on days 0, 21, and 42 [9,10,26] with human cartilage PG emulsified in the synthetic adjuvant dimethyl dioctadecyl ammonium bromide (DDA) (Sigma-Aldrich, St. Louis, MO, USA). The paws of mice, including the ankle and wrist joints, were inspected for signs of arthritis (swelling and redness) twice a week after the third immunization. The degree of arthritis was scored visually on a scale of 0 to 4 for each paw (0, no swelling or redness; 1, mild swelling/redness; 2, moderate swelling of the entire paw, including the ankle; 3, severe swelling; 4, severe swelling with hardening of the periarticular soft tissue). Severity was expressed as a sum of inflammation scores (0 to 16 per mouse) as described [9,15]. Collection of human osteoarthritc cartilage (for PG isolation) from consenting patients who had undergone joint replacement surgery was approved by the Institutional Review Board of Rush University Medical Center (Chicago, IL, USA). Likewise, all experiments involving animals were reviewed and approved by the Institutional Animal Care and Use Committee of Rush University Medical Center.

### Cell separation, labeling, and transfer for imaging studies

Cells were harvested under aseptic conditions from the spleens and JDLNs (including the brachial, axillary, inguinal, and popliteal LNs) of BALB/c mice with severe arthritis in at least two paws. After hypotonic lysis of erythrocytes from the spleen cell preparations, spleen and JDLN cells were combined. T-cell enrichment was done using Abs against non-T cell populations, followed by immunomagnetic removal of the Ab-tagged cells (StemCell Technologies, Vancouver, BC, Canada). The purity of enriched T cells, assessed by flow cytometry, was typically 95% or greater. Non-T cells, which consisted mostly of B cells (serving as Ag-presenting cells [APCs] upon transfer into SCID mice) [13,27], were prepared by immunomagnetic removal of T cells (StemCell Technologies) from the donor population, resulting in less than 5% T-cell contamination. Donor T cells were labeled with a red fluorescent CellTracker dye (CMTPX; Molecular Probes, now part of Invitrogen Corporation, Carlsbad, CA, USA). Non-T cells (APCs) either were left unlabeled or were labeled with the green fluorescent CellTracker dye CMFDA (Molecular Probes) [15]. Red fluorescent T cells were mixed with non-T cells at 1:1 to 1:3 ratios and injected intravenously into SCID mice (2 × 10^7 ^cells per mouse). Ag (50 μg of human PG [hPG] without adjuvant) was also injected intraperitoneally at the time of cell transfer to ensure *in vivo *re-stimulation of donor cells [13,15]. Migration of fluorescent cells to the ankle joint or to both the ankle and the ankle-draining popliteal LN was monitored by *in vivo *TPM, using SCID mice that received labeled donor cells 2 to 4 hours or 1, 2, 3, 4, 7, 12, or 18 days before imaging (3 to 8 mice per time point). To visualize the entry of freshly isolated and labeled T cells into already inflamed joints, some SCID mice were injected first with unlabeled donor cells. After the hindpaws became arthritic, these mice received a second transfer of CellTracker-labeled donor cells, and the migration of fluorescent cells to the inflamed ankles and the popliteal LNs was monitored by TPM. In the case of EGFP-LysM KI BALB/c mice, which express the green fluorescent protein at high levels in neutrophils [25], only the ankle joint was subjected to TPM upon the development of PGIA.

### *In vivo *two-photon microscopy

Deep-tissue imaging of the ankle joints and popliteal LNs was performed using the Prairie Ultima two-photon imaging system (Prairie Technologies, Middleton, WI, USA). Before TPM, the mouse was anesthetized with a mixture of xylazine and ketamine, and the hindlimb was fastened to the bottom of a large-volume heated imaging chamber (Bioscience Tools, San Diego, CA, USA) using veterinary-grade super glue and adhesive strips. The skin covering the lateral side of the ankle and the popliteal area was surgically excised under a stereo microscope. With a small cut on the fat tissue in the popliteal region, the popliteal LN was brought to the surface and held in place with a clamp applied to the surrounding fat and muscle. Bleeding from the cuts was modest and was stopped by cauterization. The imaging chamber was filled with warm (37°C) saline and transferred to the microscope stage. The body of the mouse was placed on a heated pad (Fine Science Tools, Foster City, CA, USA), and the ankle or LN was exposed to the water-immersion objective (× 40; numerical aperture 0.8) of an upright Olympus BX51WI microscope (Olympus USA, Center Valley, PA, USA). The temperature of both the imaging chamber and the microscope objective (wrapped in an objective heater) was kept constant (37°C) by programmable temperature controllers (Bioscience Tools). Anesthesia was maintained by repeated injection of anesthetics (for short-term imaging sessions) or by inhalation of isoflurane with oxygen (for imaging sessions lasting several hours), using a rodent inhalation anesthesia system (Protech International Inc., Boerne, TX, USA). The two-photon laser (Chameleon Ultra; Coherent Inc., Santa Clara, CA, USA) was tuned to an excitation wavelength of 820 nm for two-color imaging or 807 nm for three-color acquisition. Fluorescence emission was separated by three filter cubes, each containing a dichroic mirror and an appropriate set of filters (435 to 485 nm for blue, 500 to 550 nm for green, and 570 to 625 nm for red fluorescence) [28]. Emitted fluorescent light was detected by photomultiplier tubes (Hamamatsu, Hamamatsu City, Japan). A stage motor was used to move the specimen in x, y, z directions, and serial images were generated by axial (z) slicing in 1- to 5-μm increments (up to 300 μm deep into the tissue). Images (usually 512 × 512 pixels, 0.589 μm/pixel) were captured by PrairieView software (Prairie Technologies). Since the capture of two-color images was faster than the capture of three-color images, we routinely used two channels for image acquisition in SCID mice transferred with CellTracker Red-labeled T cells along with unlabeled non-T cells. Three-color acquisition was employed for simultaneous visualization of CellTracker Red-labeled T cells and co-transferred CellTracker Green-labeled APCs. In each case, one channel was used for visualization of the 'tissue context' (for example, endogenous fluorescence from connective tissue collagen) [29]. Image editing and three-dimensional and four-dimensional rendering were performed using either MetaMorph (Molecular Devices Corporation, Sunnyvale, CA, USA) or Imaris (version 6.1.3; Bitplane, Saint Paul, MN, USA) image processing and analysis software.

### FTY720 treatment

For treatment studies, cells were combined after isolation from the spleens and JDLNs of arthritic donors but were not subjected to any separation or labeling. These cells ('complete' donor population) were injected intravenously into SCID mice (2 × 10^7 ^cells per mouse). In the case of T cell-depleted transfer ('negative control' groups), T cells were removed from the same population of donor cells by immunomagnetic separation, and the remaining non-T cells were injected intravenously into the SCID hosts (2 × 10^7 ^cells per mouse). Although a number of SCID mice receiving complete populations of donor cells developed arthritis beginning on day 8 or 9 after transfer, we injected them once again with the same number and same type (complete or T cell-depleted) of donor cells between days 15 and 35 to achieve 100% disease incidence. All cell transfers were accompanied with intraperitoneal injection of hPG without adjuvant. Cell recipient SCID mice were inspected for arthritis symptoms every second or fourth day from day 8 and scored for disease severity as described for the donor BALB/c mice.

SCID mice were administered FTY720 (Cayman Chemical Company, Ann Arbor, MI, USA) via gavage at a dose (1 mg/kg) reported to have a therapeutic effect in autoimmune disease models [23,30]. FTY720 was administered daily on the first 3 days following the first transfer of complete donor cell populations and every second day afterwards. Control mice also received complete cell transfers and were fed with 'placebo' (5% ethanol in water). The second control group of SCID mice (transferred twice with T cell-depleted donor populations) did not receive any other treatment. In separate experiments, immunocompetent (wild-type) BALB/c mice were fed with placebo or FTY720 under the same dosing regime, beginning 1 week after the last PG injection (short-term treatment, lasting for 4 weeks) or beginning on the day of the first PG immunization (long-term treatment, lasting for 10 weeks). Blood samples were collected weekly from the facial veins by means of sterile lancets, and changes in peripheral leukocyte subsets (T and B cells and granulocytes) were monitored by flow cytometry.

### Histology and immunohistochemistry

The hindlimbs of mice were dissected, fixed in 10% buffered formalin, decalcified, and embedded in paraffin. Serial sections (6 μm thick) were cut, stained with hematoxylin and eosin, and examined under a Nikon Microphot bright field microscope (Nikon, Melville, NY, USA). Histology images were prepared using a digital color CCD (charge-coupled device) camera (CoolSnap; Photometrics, Tucson, AZ, USA) and MetaMorph software. For frozen sections, hindpaws and JDLNs of SCID mice (transferred with red fluorescence-labeled T cells and unlabeled non-T cells) were embedded in OCT compound and snap-frozen. Sections (8 μm thick) were cut on a MICROM HM 550 cryostat (MICROM International, Walldorf, Germany) and stored at -20°C until use. Cryosections were fixed in cold acetone and blocked with 5% normal goat serum and 5 μg/mL anti-CD16/32 monoclonal antibody (mAb) (Fc Block; BD Biosciences, San Jose, CA, USA) in phosphate-buffered saline (PBS). Sections were then probed with Alexa Fluor 488-conjugated mAbs against CD3, CD4, or Gr-1 (BD Biosciences or eBioscience, San Diego, CA, USA). Following post-fixation with 10% formalin, fluorescent cells within the sections were visualized using TPM.

### Cell harvest for flow cytometry

Blood samples were collected in heparin-containing tubes, and red blood cells were eliminated by hypotonic lysis. The white blood cell pellet was washed and processed for flow cytometry as described below. Single-cell suspensions were prepared separately from the spleens and JDLNs of donor cell-reconstituted SCID mice at the end of FTY720 treatment experiments. Synovial fluid was harvested post-mortem from arthritic ankles by puncturing of the lateral side of the joint with a syringe needle. The punctured joints were subjected to gentle pressure, and the released synovial fluid was pipetted into Ca^2+^-Mg^2+^-free PBS. Blood-contaminated synovial fluid samples were discarded. Synovial fluid cells were also collected from the non-arthritic ankles of SCID mice (which received T cell-depleted transfer) by joint lavage. However, these joint fluid samples contained very few cells, and lavage fluid (pooled from 8 to 10 ankles at a time) did not yield enough cells for a reliable measurement of the cellular composition by flow cytometry. Occasionally, cells were also isolated from the synovial tissue, excised from inflamed ankles, by digestion with 1 mg/mL collagenase D (Roche Diagnostics, Indianapolis, IN, USA) at 37°C for 1 hour. Fc receptors on leukocytes in the blood, spleen, JDLN, and synovial cell samples were blocked with Fc Block prior to the specific staining. Immunostaining was performed using fluorescence-conjugated mAbs against CD45, CD3, CD4, and B220 and occasionally against Gr-1 and CD11b (mAbs from BD Biosciences or eBioscience). Flow cytometry was performed using a BD FACS Canto II instrument, and data were analyzed with FACS Diva software (version 5.0) (BD Flow Cytometry Systems, San Jose, CA, USA).

### *In vitro *assays of proteoglycan-specific T-cell responses

These assays were performed as described before [9,10,15]. In brief, spleen cells were harvested under aseptic conditions and cultured in 96-well plates at a density of 3 × 10^5 ^cells per well in Dulbecco's modified Eagle medium containing 10% fetal bovine serum in the presence or absence of hPG (25 μg/mL) as Ag (triplicate wells for each treatment). Half of the supernatant was collected for interleukin-2 (IL-2) measurement on day 2 and replaced with fresh culture medium (non-stimulated control) or with medium containing PG. Cells were cultured for 6 days, and [^3^H]thymidine (0.5 μCi/well) was added for the final 16 hours of culture. Cells were harvested using an automated harvester (FilterMate; PerkinElmer, Waltham, MA, USA), and isotope incorporation into DNA was measured with a scintillation counter (MicroBeta; PerkinElmer). PG-specific cell proliferation results were expressed as stimulation index (SI) (a ratio of isotope incorporation by PG-stimulated and non-stimulated cultures). The supernatants from day 2 cultures were incubated with IL-2-dependent CTLL-2 cells (American Type Culture Collection, Manassas, VA, USA), and CTLL-2 proliferation was determined by [^3^H]thymidine incorporation, as described for spleen cells. CTLL-2 cell proliferation in the presence of bioactive IL-2, produced by PG-stimulated cultures relative to non-stimulated cultures, was expressed as SI.

### Measurement of serum proteoglycan-specific antibodies by ELISA

Serum concentrations of PG-specific Abs from the different treatment groups of SCID mice were determined by enzyme-linked immunosorbent assay (ELISA) as described [9,10,15]. Briefly, MaxiSorp ELISA plates (Nunc, Roskilde, Denmark) were coated with 0.75 μg/well of hPG or 1 μg/well of mPG overnight. Unbound material was washed out, and the wells were blocked with 1.5% fat-free milk in PBS. Serially diluted (1:100 to 1:200,000) serum samples from individual mice and internal standard samples (serum pooled from arthritic BALB/c mice, containing known amounts of PG-specific IgG1 and IgG2a) were incubated with the immobilized PG. hPG- or mPG-specific IgG1 (or IgG2a) was detected using horseradish peroxidase (HRP)-conjugated secondary Abs (Invitrogen Corporation), followed by HRP substrate and *o*-phenylene-diamine (Sigma-Aldrich) as chromogen. Optical densities were measured at 490 nm using a Synergy 2 ELISA reader (BioTek Instruments, Winooski, VT, USA). Results were expressed as milligrams or micrograms of PG-specific IgG/mL serum.

### Statistical analysis

Statistical analysis was performed using SPSS software (version 16; SPSS Inc., Chicago, IL, USA). Depending on the homogeneity of variance, data were analyzed directly or were transformed prior to analysis. Data from two groups were compared using the independent samples Student *t *test (two-tailed), and multiple group comparisons were made using analysis of variance with the *post hoc *Dunnett *t *test. *P *values of 0.05 or less were accepted as statistically significant.

## Results

### *In vivo *and *ex vivo *imaging methods reveal poor T-cell migration into the joints during the adoptive transfer of PGIA to SCID mice

**Figure 1:**
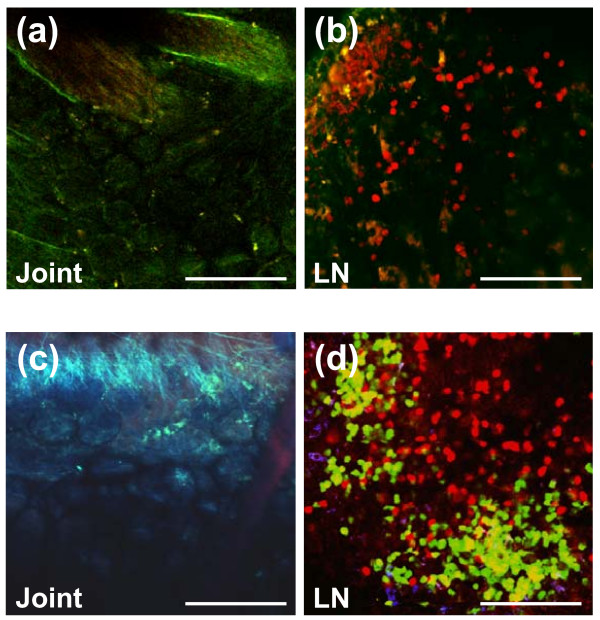
**T cells or B cells, transferred from arthritic BALB/c mice to severe combined immunodeficient (SCID) mice, are detectable by *in vivo *imaging in the popliteal lymph nodes (LNs) but not in the joints of the recipient mice**. **(a) **Two-photon microscopy (TPM) image of the ankle joint of a SCID mouse 2 days after transfer of CellTracker Red (CMTPX)-labeled T cells and unlabeled non-T cells (antigen-presenting cells, or APCs) from arthritic BALB/c donors. No red fluorescent cells are visible in the joint. Second harmonic generation (SHG) signals from collagen fibers (around blood vessels) are detected in the green fluorescence channel in two-color acquisition. **(b) **TPM image of the joint-draining (popliteal) LN from the same mouse shows numerous red fluorescent donor T cells that homed to the LN. SHG (green) signals are from collagen in the LN capsule and stroma. **(c) **TPM image of the inflamed ankle of a SCID mouse 12 days after transfer of CellTracker Red-labeled T cells and CellTracker Green (CMFDA)-labeled APCs (>85% B cells) from arthritic donors. Although this joint was heavily inflamed, no red fluorescent T cells (or green fluorescent B cells) were found by *in vivo *TPM imaging. SHG signals from collagen are detected in the blue channel in this three-color acquisition image. Endogenous (auto) fluorescence from macrophages appears in light green. **(d) **TPM image of the popliteal LN from the same mouse shows large numbers of red fluorescent T cells and green fluorescent non-T cells (mostly B cells), which occupy the T-cell and B-cell zones of the LN, respectively. The TPM images shown are representative samples of ankle and LN images from six SCID mice at each time point. Scale bars, 100 μm.

Following intravenous injection of a mixture of CMTPX (red fluorescent dye)-labeled T cells and unlabeled non-T cells (APCs) or of CMTPX-labeled T cells and CMFDA (green fluorescent dye)-labeled APCs from arthritic BALB/c to SCID mice, we used TPM to monitor donor cell recruitment in the ankle joints of the recipients 1, 2, 3, 4, 7, 12, and 18 days after cell transfer. We were unable to detect T cells in a consistent manner in the ankle joints of SCID recipients using TPM imaging (Figure 1a, c). As expected, transferred red fluorescent T cells (Figure 1b) or both red T cells and green non-T cells (Figure 1d) were found in the ankle-draining popliteal LNs at both earlier (day 2, Figure 1b) and later (day 12, Figure 1d) time points. The SCID mouse (whose joint and LN images are shown in the bottom panels of Figure 1) already had arthritis in the imaged ankle; however, no T cells were visible; only autofluorescent macrophages (light green) and second harmonic generation signals from collagen fibers (blue) [29] were detected in the synovial tissue (Figure 1c).

The virtual absence of donor T cells in the SCID joints was not due to technical problems with fluorescent cell detection in the ankle by TPM given that both CMTPX- and CMFDA-labeled cells could be visualized if injected directly into the joint (Figure S1a in Additional file 1). Moreover, green fluorescent neutrophil granulocytes were easily detected in the ankles of EGFP-LysM KI BALB/c mice upon induction of PGIA (Figure S1b in Additional file 1). In the SCID transfer experiments, a donor cell occasionally could be seen moving in the synovial blood vessels of the recipient at early time points (up to 1 day) after injection of red CMTPX-labeled unseparated (Figure S1c in Additional file 1) or T cell-enriched (Figure S1d,e in Additional file 1) donor populations. When judged on the basis of shape, motile behavior [31], or exclusion of cytoplasmic fluorescent dye by lobulated nuclei (Figure S1e in Additional file 1), such cells appeared to be neutrophils rather than lymphocytes. The spleens of arthritic donor mice contain only a small population (up to 6%) of neutrophils, but these cells are subject to preferential recruitment in synovial vessels as compared with lymphocytes [15,31]. Since transferred neutrophils do not live long in the recipients, donor cells visualized several days after transfer (Figure S1f in Additional file 1) could be lymphocytes. However, the frequency of donor cell appearance in the SCID joints seemed to decrease further with time.

In contrast to a poor recruitment to the joints, red fluorescent T cells and green fluorescent non-T cells (mostly B cells) migrated in large numbers to the popliteal LNs and occupied their respective territories (Figure 1d). The frequency of donor cells visualized in the LN did not seem to decrease with time, suggesting that intracellular fluorescence did not fade significantly during the 18-day time frame of TPM monitoring. Most of the cells in the LN showed a polarized shape and moved around vigorously during the imaging sessions (Supplemental video 1 in Additional file 2), as reported by others using TPM to reveal lymphocyte motility in mouse LNs [28,32-34].

**Figure 2:**
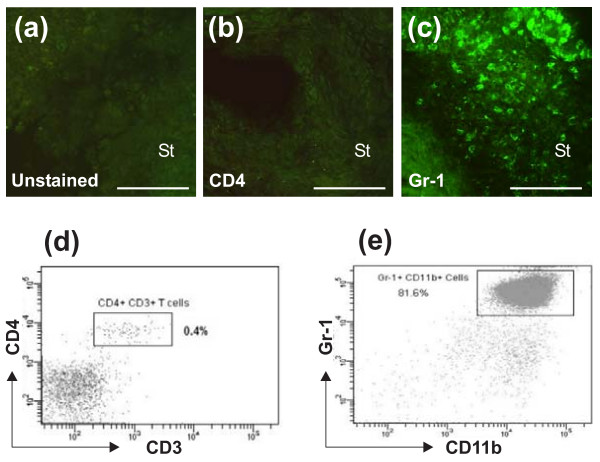
**Immunohistochemical and flow cytometric detection of T cells and granulocytes in inflamed ankle joints of severe combined immunodeficient (SCID) mice with adoptively transferred proteoglycan-induced arthritis (PGIA)**. The SCID mouse used for immunohistochemistry developed arthritis 9 days after receiving unlabeled cell transfer and was re-transferred with CellTracker Red-labeled T cells and unlabeled non-T cells on day 10. *In vivo *two-photon microscopy imaging of the inflamed ankle on day 12 revealed no red fluorescent cells. The mouse was sacrificed, and frozen sections were prepared from the ankle after imaging. **(a) **No red fluorescent T cells are visible in the unstained section of the inflamed ankle. **(b) **Immunostaining with green fluorophore-conjugated anti-CD4 monoclonal antibody (mAb) shows no evidence of CD4^+ ^T helper cells (no yellow or green color). **(c) **Anti-Gr-1 mAb against granulocytes stains numerous cells in the same joint. Scale bars, 100 μm. St, synovial tissue. **(d) **Flow cytometry of synovial fluid cells from the arthritic ankle joints of a SCID mouse shows a small population of T cells, nearly all of which are CD4^+^, in the fluid. **(e) **Synovial fluid from arthritic SCID ankles contains a large proportion of Gr-1^hi ^granulocytes that also express high levels of CD11b. Flow cytometry was done using synovial fluid samples from SCID mice that developed PGIA approximately 2 weeks after transfer of unlabeled cells from arthritic donors. Flow data are representative of at least six synovial fluid samples harvested from inflamed ankle joints of SCID mice with adoptively transferred PGIA.

To further investigate whether some T cells (not detected by *in vivo *TPM) were present in deeper areas of the joints of fluorescent donor cell-injected SCID mice, we prepared serial cryosections from non-arthritic or arthritic ankles of these mice following TPM imaging. The sections were left unstained (to visualize red fluorescent T cells) or were immunostained for T cells with a green fluorescent mAb against CD3 or CD4. Again, we were not able to detect red fluorescent cells (unstained section, Figure 2a) or CD3^+ ^(not shown) or CD4^+ ^(Figure 2b) cells in these joints (sections of inflamed synovial tissue, shown in Figure 2, are from a SCID mouse with adoptively transferred acute arthritis in the ankle). In contrast, anti-Gr-1 staining of sections of arthritic joints gave strong signals (Figure 2c), indicating that the majority of infiltrating cells were granulocytes (neutrophils) in the inflamed joint, as reported previously [15,31], and neutrophils were also in the arthritic ankle of an EGFP-LysM KI mouse (Figure S1b in Additional file 1). Next, we asked whether T cells in the synovial fluid of inflamed ankles of SCID mice were detectable by flow cytometry. Immunostaining of synovial fluid cells for CD3 and CD4 and subsequent flow cytometry revealed the presence of a small population of T lymphocytes (mostly CD4^+^), comprising less than 1% of the cells present in the joint fluid of arthritic ankles (Figure 2d). The number of T cells was even less (essentially negligible) when collagenase-digested synovial tissue samples were assayed by flow cytometry (data not shown). As in the case of IHC, the dominant cell population in synovial fluid of SCID ankles was found to be Gr-1^hi ^neutrophils that also expressed high levels of CD11b/Mac-1 (Figure 2e), the integrin found on leukocytes of myeloid lineage [35].

### Limiting T-cell access to the joints by FTY720 treatment after cell transfer does not inhibit arthritis development in SCID mice, but removal of T cells before transfer does

The presence of a small population of T cells in the synovial fluid after the development of adoptive PGIA compelled us to investigate whether the few T cells present in the joints played some role in the local inflammatory process. If so, blockade of T-cell entry from the bloodstream into the joint could prevent or suppress inflammation. To this end, we chose to administer oral treatment with the S1P receptor modulator FTY720 [20] to the SCID mice during the adoptive transfer of PGIA. The principal mechanism of action of FTY720 is the induction of internalization of S1P receptors (including S1P_1_/S1PR1, which is highly expressed on T cells circulating in blood and lymph) with subsequent loss of cell response to S1P [21]. S1P has been shown to direct T-cell egress from lymphoid organs [21]. Therefore, treatment of animals with the S1P 'agonist' FTY720 or genetic deletion of S1P_1_/S1PR1 renders these animals lymphopenic [20,21], thereby preventing the entry of lymphocytes (primarily T cells) into peripheral organs. The immunosuppressive effect of FTY720 in some autoimmune disease models, as well as in human MS, has been attributed primarily to peripheral T-cell depletion [23,24,30]. It was expected, therefore, that if the modest population of joint-homing T cells had a local pro-inflammatory role in the development of adoptive PGIA in SCID mice, limiting their access to the joints could inhibit inflammation.

**Figure 3:**
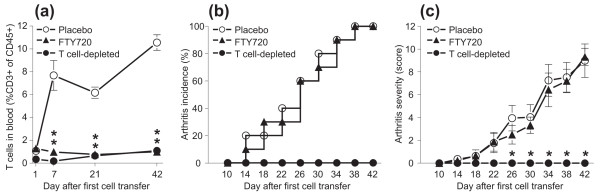
**Effects of FTY720 treatment or depletion of T cells from the transferred donor cells on circulating T cells and arthritis development in severe combined immunodeficient (SCID) mice**. SCID mice, injected with lymphocytes from arthritic donors, were subjected to treatment with placebo (open circles) or FTY720 (closed triangles), as described in Materials and Methods. A separate group of SCID mice received cells from the same arthritic donors, but T cells from the donor population were depleted prior to transfer (solid circles). **(a) **The proportion of T cells (CD3^+^) among all blood leukocytes (CD45^+^) was monitored by flow cytometry from between day 1 after the first cell transfer and day 42 (end of experiment). Data shown are the mean ± standard error of the mean (SEM). (n = 9 to 11 mice per group; *P < 0.01 in comparison with the placebo-treated group.) **(b) **The SCID mice were inspected for arthritis symptoms at 4-day intervals between days 10 and 42. Incidence of proteoglycan-induced arthritis is expressed as the percentage of arthritic animals among all SCID mice in the respective groups. **(c) **The degree of inflammation in each paw was scored visually at 4-day intervals. Arthritis severity is expressed as the mean ± SEM of cumulative paw scores (n = 9 to 11 mice per group; *P < 0.01 in comparison with both the placebo- and FTY720-treated groups).

The control (placebo-treated) and FTY720-treated groups of SCID mice received complete cell transfer (both T and non-T cells) from arthritic donors, and a second control group received cells from the same donors, but from which the T cells had been depleted prior to transfer [13]. As shown in Figure 3a, T cells expanded in peripheral blood in the placebo-treated SCID recipients but not in the FTY720-treated SCID mice; both sets of mice received complete cell transfer. In SCID mice transferred with T cell-depleted donor populations (Figure 3a), T cells were barely detectable in the blood 7 days after the first transfer, but some T cells emerged in the circulation with time. This was most likely due to homeostatic expansion of the few T cells ('contaminants' in the T-depleted cell fractions) in the lymphopenic environment [13], some of which were released into blood. However, the peripheral T-cell pool in the T-depleted transfer groups was as small in size as the corresponding pool in the FTY720-treated mice from day 21 after cell transfer (Figure 3a). Neither FTY720 treatment nor depletion of donor T cells prior to transfer had a strong negative impact on the percentage of circulating B cells or granulocytes (data not shown). Surprisingly, although FTY720 treatment kept the proportion of blood T cells very low (approximately 1% of all CD45^+ ^leukocytes, Figure 3a), it did not prevent or delay the onset of adoptive PGIA (Figure 3b). Placebo- and FTY720-treated SCID mice developed arthritis with similar kinetics, and both groups achieved 100% disease incidence within 6 weeks after the first cell transfer. In contrast, SCID hosts transferred with T cell-depleted donor populations, despite having as many circulating T cells as the FTY720-treated mice from day 21 (Figure 3a), did not develop disease at all (Figure 3b, c). FTY720 also failed to suppress arthritis severity as the disease scores were similar in the groups treated with placebo and FTY720 (Figure 3c).

### FTY720 treatment has no effect on the development of primary PGIA in immunocompetent BALB/c mice

**Figure 4:**
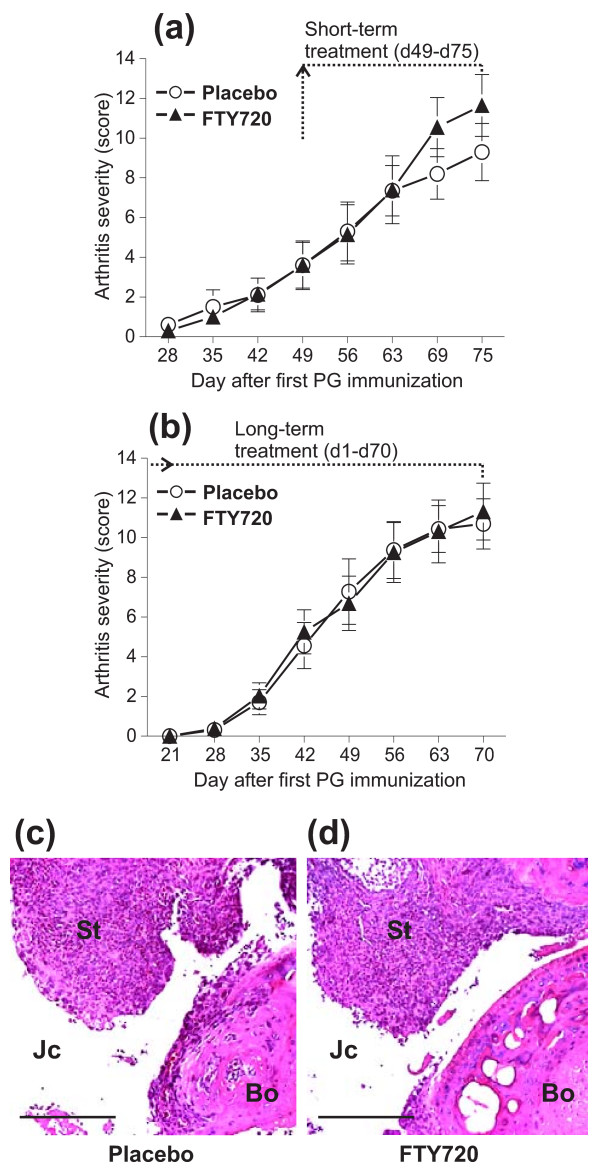
**Treatment of immunocompetent (wild-type) BALB/c mice with FTY720 has no effect on the development of the primary form of proteoglycan (PG)-induced arthritis**. **(a) **Short-term treatment with placebo (open circles) or FTY720 (closed triangles) started on day 49 or 50 (1 week after the third PG immunization) and ended on day 75. **(b) **Long-term (prophylactic) treatment was initiated after the first immunization and ended on day 70. Data shown are the mean ± standard error of the mean of cumulative arthritis scores over time (short-term treatment, n = 10 mice per group; long-term treatment, n = 16 mice per group; the difference between placebo- and FTY720-treated groups was not significant in either case). **(c) **Histology of the ankle joint of a placebo-treated mouse from the long-term treatment group. **(d) **Histology of the ankle of an FTY720-treated mouse from the long-term treatment group. Sagittal sections of decalcified and paraffin-embedded joints were stained with hematoxylin and eosin. The degree of synovial tissue hyperplasia, leukocyte infiltration, or cartilage erosion was similar in both joints. Scale bars, 250 μm. Bo, bone (talus); Jc, joint cavity; St, synovial tissue.

To determine whether FTY720 was also ineffective in suppressing or preventing arthritis in immunocompetent BALB/c mice, we administered placebo or FTY720 orally to BALB/c mice after immunizing them with PG in DDA adjuvant to induce the primary form of PGIA. Short-term treatment groups received placebo or FTY720 every second day from day 49 or 50 (1 week after the third PG injection) through day 75, and long-term treatment was employed from the first PG immunization (day 1) through day 70. In both cases, FTY720 quickly and significantly depleted T cells in the circulation (by greater than 90% from the first week through the end of the treatment period). However, no delay in arthritis development was observed in the FTY720-treated groups in either case, and the arthritis scores were not significantly different between placebo-treated and FTY720-treated mice that had undergone either short-term (Figure 4a) or long-term (Figure 4b) treatment. Consistent with the similar disease onset times and scores, histopathology of the ankle joints on day 70 showed comparably high degrees of leukocyte infiltration, synovial hyperplasia, and joint tissue destruction in mice treated with placebo (Figure 4c) and those treated with FTY720 (Figure 4d) in the long-term experiments.

### FTY720 treatment does not have an effect on the occupancy of lymphoid organs by transferred T cells

**Figure 5:**
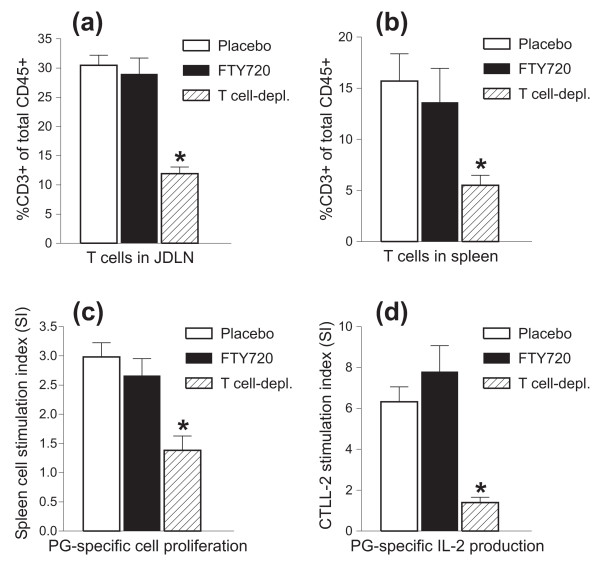
**Proportion of lymphoid organ-homing T cells and magnitude of antigen (Ag)-specific T-cell responses in severe combined immunodeficient (SCID) mice treated with placebo or FTY720 after receiving complete donor lymphocyte transfer (T cells along with non-T cells as Ag-presenting cells) and in SCID mice receiving T cell-depleted transfer**. T-cell proportions in the **(a) **joint-draining lymph nodes (JDLNs) and **(b) **spleens were measured by flow cytometry and expressed as percentage of CD3^+ ^of total CD45^+ ^cells. Data shown are the mean ± standard error of the mean (SEM) (n = 9 to 11 mice per group; *P < 0.05). **(c) **Ag (human proteoglycan)-specific spleen cell proliferation and **(d) **Ag-specific production of bioactive interleukin-2 (IL-2) by the same spleen cell cultures were assayed on the basis of [^3^H]thymidine incorporation into splenocytes and CTLL-2 cells, respectively. Results are expressed as stimulation index and are the mean ± SEM (n = 4 to 5 mice per group; *P < 0.01). Isotope incorporation by non-stimulated cell cultures was less than 1,700 cpm per well and was comparable among the three groups of mice. PG, proteoglycan.

The results of the FTY720 treatment studies suggested that the availability of circulating T cells was perhaps not crucial for arthritis development. This was the most obvious in the case of adoptive transfer experiments, in which arthritic SCID hosts, reconstituted with complete donor populations and receiving FTY720 treatment, had the same blood T-cell pool size (from day 21) as the non-arthritic hosts transferred with T cell-depleted donor fractions (Figure 3). Examination of the cellular composition of JDLNs and spleens of SCID mice at the conclusion of the transfer experiments (days 42 to 45) revealed T-cell pools of comparable size in the lymphoid organs of mice (after reconstitution with complete donor cell fractions) treated with placebo and those treated with FTY720 (Figure 5a, b). This finding was consistent with the observation that FTY720 inhibits T-cell egress from lymphoid organs but has no significant impact on the occupancy of these organs by T cells [20,21]. As expected, the T-cell pool in the lymphoid organs of SCID mice, transferred with T-depleted fractions from the same donors, was significantly reduced (Figure 5a, b). This indicated that a very small number of T cells was transferred initially, despite their subsequent homeostatic expansion.

Through blockade of T-cell exit from the lymphoid organs, FTY720 was expected to limit T-cell access to the joints. Indeed, we found the proportion (percentage) of joint fluid T cells in the inflamed ankles of FTY720-treated animals to be approximately half the percentage of T cells present in the joints of placebo-treated mice (FTY720-treated: median 0.2%, range 0.0% to 0.5%; placebo-treated: median 0.4%, range 0.2% to 0.7%; n = 6 synovial fluid samples per group), although this difference did not reach significance (*P *= 0.17). However, the degree of inflammation was similar in the joints of FTY720-treated and placebo-treated mice (also, see Figure 3c).

### FTY720 treatment does not reduce proteoglycan-specific T-cell responses or serum autoantibody levels, while pre-transfer depletion of T cells completely inhibits autoantibody production

Next, we asked whether Ag (PG)-specific T- or B-cell responses were compromised by FTY720 treatment. As shown in Figure 5c, proliferation of spleen T cells in response to *in vitro *PG stimulation was comparable in the placebo-treated and FTY720-treated groups but was significantly reduced in the T cell-depleted transfer group. Similarly, PG-specific IL-2 production (as measured by proliferation of IL-2-sensitive CTLL-2 cells) was not impaired by FTY720 treatment but was significantly reduced in the spleen cell cultures of SCID mice receiving T cell-depleted donor fractions (Figure 5d). The reduced Ag-specific spleen T-cell responses in this group of mice (Figure 5c, d) seemed to correlate directly with the low number of T cells in the spleen (Figure 5b).

**Figure 6:**
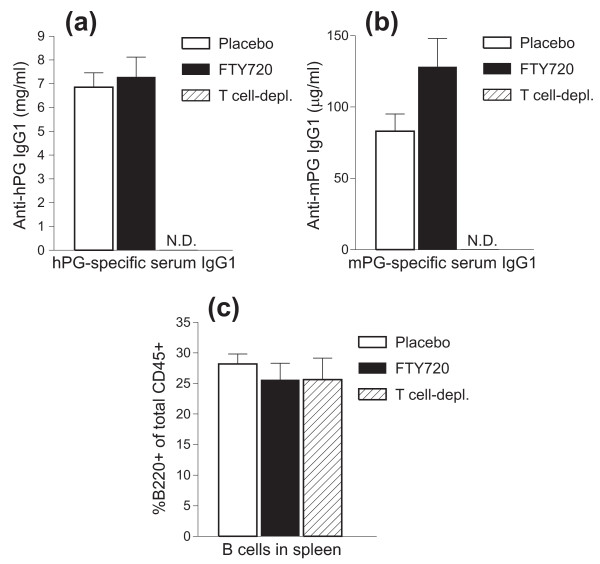
**Concentrations of proteoglycan (PG)-specific serum antibodies and percentages of spleen B cells in severe combined immunodeficient (SCID) mice treated with placebo or FTY720 after receiving complete donor lymphocyte transfer and in SCID mice receiving T cell-depleted transfer**. Serum levels of **(a) **human PG (hPG)-specific antibodies and **(b) **mouse PG (mPG)-specific autoantibodies of the IgG1 isotype were determined by enzyme-linked immunosorbent assay and expressed as milligrams per milliliter and micrograms per milliliter, respectively. N.D., not detectable. **(c) **The proportion of B cells in the spleens of the three groups of SCID mice was measured by flow cytometry and expressed as percentage B220^+ ^of total CD45^+ ^cells. Data shown are the mean + standard error of the mean (n = 9 to 11 mice per group).

To determine whether FTY720 treatment had any effect on Ab production, we compared serum concentrations of hPG-specific Abs and mPG-specific autoAbs in the three groups of mice after termination of these experiments (day 42 or 45). We found that SCID mice fed with placebo or FTY720 had similar levels of IgG1 Abs against the immunizing Ag (hPG) (Figure 6a) and that the concentration of mPG-specific autoAbs was even slightly elevated in the FTY720-treated group (Figure 6b). However, these Abs were completely absent in the sera of T-depleted donor cell recipients (Figure 6a, b; N.D.: not detectable). This was also the case when serum samples from an additional set of similar SCID transfer groups were assayed 67 days after the first cell transfer, indicating that the appearance of PG-specific Abs in serum was not simply delayed in the T cell-depleted transfer recipients (data not shown). Measurement of serum PG-specific Abs of the IgG2a isotype, which were present in much smaller amounts, revealed a similar trend, and IgG2a Abs were also absent in serum samples of the T cell-depleted transfer group (not shown). Since B cells were found in similar proportions in the spleens of all three groups of SCID mice (Figure 6c) as well as in the JDLNs (data not shown), the absence of PG-specific Ab output in the T cell-depleted transfer group could not be explained by a reduced B-cell pool in the lymphoid organs of these mice.

## Discussion

Autoimmune diseases are initiated and mediated by autoreactive T cells that can mount a direct attack on the target tissues or act in concert with B cells by providing help for the production of pathogenic autoAbs or both. In animal models of MS, for example, massive invasion of the central nervous system by 'encephalitogenic' T cells has been demonstrated by different methods, including *in vivo *imaging [18,19]. Several laboratories reported the presence of CD4^+ ^T cells in the inflamed joints in various animal models of RA, but few studies commented on the small size of this population relative to other leukocytes infiltrating the joints [14,15,31,36]. CD4^+ ^cells (both T helper 1 [Th1] and Th17 phenotypes) are present in the rheumatoid synovial tissue and these cells are also found in smaller numbers in the synovial fluid of affected joints of RA patients [3,17,37,38]. On the basis of the presence of T cells within tissue lesions in almost all forms of autoimmune diseases, it is believed that substantial influx of activated (effector) T cells into target organs is necessary for the initiation and progression of disease. Therefore, the initial aim of this study was to monitor the migration of fluorescence-labeled 'arthritogenic' T cells into the joints of mice during the adoptive transfer of PGIA, employing *in vivo *deep-tissue imaging with TPM, used for the first time in an autoimmune model of RA. In anticipation of increased T-cell traffic to the joints around the time of arthritis onset [39], we subjected the ankles of SCID recipient mice to TPM imaging at several time points prior to and after disease development. We were surprised to find that this powerful imaging technique was unable to consistently reveal the presence of T cells within the SCID joints at any stage of arthritis transfer. We could detect a small population of T cells (nearly all CD4^+^) in the synovial fluid of the arthritic joints of mice by flow cytometry. The slight discrepancy between TPM imaging and flow cytometry in the frequency of fluorescent donor cell detection might be explained by the capacity of the flow cytometer to acquire and analyze tens of thousands of cells, whereas only a few thousand cells (dispersed within the structural components of the joint) can be scanned by TPM. Alternatively, instead of navigating in the synovial tissue, some T cells may 'flip' directly into the fluid from the rich network of blood vessels that are located underneath the synovial lining [31]. However, even in the joint fluid, T cells represented less than 1% of infiltrating leukocytes, most of which were neutrophils.

To further investigate whether limiting the access of T cells to the joints would delay or suppress arthritis, we employed oral treatment with FTY720, an S1P receptor modulator that inhibits T-cell egress from the lymphoid organs [22] and subsequent entry of these cells into target tissues via the blood circulation. This mechanism of FTY720 treatment has been postulated to account for disease suppression in animal models of MS (and in patients with MS) and autoimmune diabetes [23,24,30]. Although we found that FTY720 effectively depleted T cells in the circulation and reduced T-cell presence in the synovial fluid, it failed to inhibit the development or reduce the severity of PGIA in either the primary or the adoptively transferred form of the disease. These results do not seem to be consistent with the report by Wang and colleagues [40], in which treatment of chicken CII-immunized rats with FTY720 significantly suppressed the development of CIA. Since blood T cells were depleted to a similar extent by FTY720 in rat CIA [40] and murine PGIA (Figure 3a), the discrepancy between their study and ours can be explained only by assuming that pathogenic T cells migrate to the joints in much larger numbers and have a much greater role in the local inflammatory process in rats with CIA than in mice with PGIA. This is a distinct possibility as Wang and colleagues [40] could identify numerous CD4^+ ^cells in the inflamed joint tissue of CIA rats by IHC, whereas we could detect essentially none in adoptively transferred PGIA by means of a similar method. In murine CIA, however, Holmdahl and colleagues [14] reported that T cells constitute a minor population of joint infiltrating cells. Also, in earlier studies on CIA in DBA/1 mice ([41] and our unpublished data) and on PGIA in BALB/c mice [15,31], we found that the proportion of joint-homing T cells is low in both models, suggesting that the paucity of these cells in arthritic joints is not a unique feature of PGIA.

The role of joint-infiltrating T cells in the pathology of RA has been a matter of debate for decades. On the basis of very low T-cell cytokine levels in rheumatoid synovial samples [42] and the hyporesponsiveness of joint-infiltrating T cells to T-cell receptor stimulation [43], earlier studies have questioned the impact of synovial T cells on the local inflammatory process. More recent reports have identified 'pro-inflammatory' Th1 and Th17 cells as well as 'anti-inflammatory' regulatory T cells (Tregs) in rheumatoid joints (reviewed in [3]). Tregs have also been found in inflamed synovial fluid samples of patients with juvenile idiopathic arthritis and various forms of spondylarthropathy [44], suggesting that some T cells, such as Tregs, might be attracted to sites of inflammation, where these Tregs should have a protective, rather than a destructive, role [44]. Progression of inflammatory joint destruction in the absence of synovial T cells has been reported in an RA patient with advanced HIV infection [45], highlighting the strong involvement of T cell-independent processes and non-T cells in the joint pathology of RA. In serum or Ab transfer-induced models of RA, transient arthritis develops apparently in an (autoreactive) T cell-independent manner and is characterized by massive influx of neutrophils into the joints of naïve recipient mice [46,47]. Interestingly, a minor T-cell population was also found in the synovial fluid of naïve mice after induction of arthritis by serum transfer, and Tregs were identified as a prominent population of these joint-homing T cells [48]. As in the case of Tregs found in arthritic joints of patients with RA and other arthropathies [44], the presence of Tregs in the joints of naïve animals after passive transfer of arthritis argues for a secondary recruitment of these T cells to the site of inflammation.

Although PGIA does not appear to be inducible via serum transfer ([12] and our unpublished data), neutrophils still vastly outnumber T cells in the inflamed joints in PG-immunized arthritic BALB/c mice and in SCID mice with the adoptively transferred form of the disease [15,31]. As we demonstrate in this study, it is easy to identify Gr-1^hi ^cells, in contrast to T cells, in both synovial tissue and fluid samples of inflamed ankle joints of SCID mice with adoptively transferred PGIA. Cell depletion experiments indicate that neutrophils are directly involved in the local inflammatory and destructive processes as anti-Gr-1 mAb-mediated elimination of circulating neutrophils promptly abrogates arthritis in both PGIA [49] and a serum/Ab transfer-induced model of RA [47].

In contrast to neutrophil depletion, our study demonstrates that reduction of circulating T cells by FTY720 treatment does not have a significant effect on disease onset or severity in PGIA. Since FTY720 treatment significantly lowered the number of circulating T cells but did not completely eradicate them from the blood or from joint effusions, the conclusion that can be drawn from this part of our study is that the initiation and effector phases of PGIA are quite independent of the availability of T cells in the circulation. Transfer of arthritogenic donor cells, from which T cells had been depleted prior to injection into the SCID mice, however, did not result in arthritis, suggesting that substantial T-cell presence in the recipient's immune system (but not in the joints) was an absolute requirement for disease development.

The absence of PG-specific (including autoreactive) serum Abs in mice receiving T-depleted cell transfer, despite a B-cell pool of normal size, indicates a requirement for robust T-cell help for effective production of Ag-specific Abs by B cells. The reduced size of T-cell pool found in the lymphoid organs and the lack of circulating PG-specific Abs suggest that B cells did not receive adequate T-cell help in these mice. Furthermore, the lack of PG-specific Abs (and mPG-specific autoAbs, in particular) in the serum and the concomitant absence of arthritis in the T cell-depleted transfer groups indicate an important contribution of these Abs to disease development. Indeed, autoAbs have been shown to be pathogenic in autoimmune models of RA [27,50,51] as serum Abs against murine CII, G6PI, or other autoAgs can induce transient arthritis when injected into naïve mice [46,51,52]. Deposition of autoAg- and IgG-containing immune complexes (ICs) in the joints has been repeatedly reported in RA and serum/Ab-induced arthritis models [2,53-56]. Some Abs, which may access the joints by crossing 'leaky' synovial blood vessels [56], remain associated with autoAgs expressed locally in the joint (for example, proteins present on the surface of articular cartilage or in the synovial fluid). The role of ICs in complement fixation is well known, as is the involvement of ICs and complement fragments in the rapid recruitment of Fc receptor-bearing phagocytic cells (neutrophils and monocytes) in the joint from the circulation, leading to tissue infiltration and swelling [43,46,53,55,57]. Although neither immune serum nor T cells or B cells (from arthritic animals) appear to be capable of transferring PGIA to SCID mice when injected alone [12,13], mild and transient synovitis is observed after co-injection of immune serum and T cells (but not B cells), and this mild inflammation can even be extended by repeated administration of immune serum ([27] and our unpublished data). This observation suggests that arthritogenic Abs and T cells may briefly synergize to initiate inflammation but that continuous *in vivo *production of autoAbs by B cells (probably in a T cell-supported manner) is necessary for the perpetuation of the inflammatory process and establishment of chronic disease.

## Conclusions

Here, we show that the development of autoimmune arthritis in an animal model of RA is not accompanied by a robust influx of T cells into the joints and that inflammation is rampant even if the availability of circulating T cells is profoundly reduced. Arthritis does not develop when T-cell presence is also reduced in the secondary lymphoid organs, in which case autoAbs are not detected in the circulation. According to these observations, the major contribution of T cells to joint inflammation stems from their capacity to provide help to B cells within the lymphoid organs for systemic production of pathogenic autoAbs. Although some rare joint-infiltrating T cells may have a role in the recruitment of other leukocytes (neutrophils and monocytes), the local inflammatory process appears to be dependent on the availability of circulating autoAbs (and neutrophils) rather than T cells. As discussed above, strong dependence of arthritis on autoimmunity, but limited or no dependence on local T cells, is observed (or at least suspected) in other animal models of RA. Whether this is a paradox restricted to animal models or is also a paradox of the human disease (for example, in patients with seropositive forms of RA) remains to be determined.

## Abbreviations

Ab: antibody; Ag: antigen; APC: antigen-presenting cell; autoAb: autoantibody; autoAg: autoantigen; CIA: collagen-induced arthritis; CII: type II collagen; DDA: dimethyl dioctadecyl ammonium bromide; EAE: experimental allergic encephalomyelitis; EGFP-LysM KI: enhanced green fluorescent protein-lysozyme M knock-in; ELISA: enzyme-linked immunosorbent assay; G6PI: glucose-6-phosphate isomerase; hPG: human proteoglycan; HRP: horseradish peroxidase; IC: immune complex; IHC: immunohistochemistry; IL-2: interleukin-2; JDLN: joint-draining lymph node; LN: lymph node; mAb: monoclonal antibody; mPG: mouse proteoglycan; MS: multiple sclerosis; PBS: phosphate-buffered saline; PG: proteoglycan (cartilage aggrecan); PGIA: proteoglycan-induced arthritis; RA: rheumatoid arthritis; S1P: sphingosine 1-phosphate; SCID: severe combined immunodeficient; SI: stimulation index; Th: T helper; TPM: two-photon microscopy; Treg: regulatory T cell.

## Competing interests

The authors declare that they have no competing interests.

## Authors' contributions

AA designed and performed most of the animal experiments and drafted the manuscript. CE was involved in study design, carried out flow cytometry and TPM, and drafted the manuscript. TK performed IHC and TPM. KO carried out cell proliferation and ELISA assays. AL collected all the data and performed the statistical analyses. TTG was involved in study design and carried out the PG immunizations and histological assessment of arthritis. KM participated in study design, coordinated the experiments, and wrote the manuscript. All authors read and approved the final manuscript.

## Supplementary Material

Additional file 1 **Supplemental Figure 1 (Figure S1)**. Technical issues with in vivo joint imaging and rare cases of donor cell appearance in SCID joints after cell transfer. **(a) **Fluorescence-labeled cells injected directly into the joint cavity can be easily visualized by TPM. A mixture of CMTPX (red) and CMFDA (green)-labeled lymphocytes (~1,000 cells) was injected into the cavity of the ankle joint of a BALB/c mouse. The joint was subjected to TPM imaging 2 days after the intra-articular cell injection. Red and green fluorescent cells are visible in the joint in the vicinity of connective tissue (blue). **(b) **EGFP-expressing granulocytes (neutrophils) are abundant in the joints of EGFP-LysM KI mice with acute PGIA. Mice with EGFP knocked in the lysozyme M locus (EGFP-LysM KI), which express this fluorescent protein in their granulocytes at very high levels, were backcrossed into the BALB/c background and immunized to induce PGIA. TPM imaging was performed on the arthritic ankle joint 2 days after disease onset. Numerous green fluorescent cells are visible in the synovial tissue and cavity. **(c) **Fluorescence-labeled donor cells appear in the circulation of recipient SCID mice at early time points after transfer. Unseparated spleen-LN cells from arthritic donor mice were labeled with red CMTPX and injected i.v. into SCID recipients. TPM imaging of the ankle joint was carried out on the day following cell transfer. A single red fluorescent cell (arrow) is visible at a branching point of synovial blood vessels. Time-lapse video revealed a motility pattern that was similar to the behavior of neutrophils in EGFP-LysM KI mice, making uncertain that the red cell was a lymphocyte. **(d-f) **Donor cells are occasionally found in the synovial tissue or vessels after transfer of T cell-enriched fractions. **(d) **A red fluorescent cell (arrow) is visible in the synovial tissue 1 day after i.v. transfer of CMTPX-labeled T cells (along with CMFDA-labeled APCs) from arthritic donors to a SCID mouse. **(e) **High-magnification imaging of the midsection of the same immobile red cell (boxed in panel d) revealed the presence of cytoplasmic 'holes' corresponding to nuclear lobes. This raised the suspicion of this red cell being a polymorphonuclear leukocyte from the donor spleen cell preparation. **(f) **This SCID joint was imaged 4 days after the transfer of CMTPX-labeled T cells and unlabeled APC from arthritic donors. A single red cell (arrow) is seen within the shadow of a blood vessel. Serial images showed tethering-rolling movement of this red cell along the vessel walls, indicating that it was located intravascularly. The presence of donor cells in the recipients' joints was not typical, and was even less frequent at later time points (days 7-18) after transfer. Scale bars, 50 μm. Click here for file

Additional file 2 **Supplemental Video 1**. Localization and motility of fluorescent T and B cells in the popliteal LN of a SCID mouse following their transfer from arthritic BALB/c mice. Donor T cells were labeled with CellTracker Red (CMTPX) and non-T cells (~90% B cells) with CellTracker Green (CMFDA), and mixed at a ratio of 1:2 prior to injection into the SCID mouse. The popliteal LN was subjected to TPM imaging on day 7 after cell transfer. Z series of images were acquired from the LN in vivo. Autofluorescent subcapsular sinus macrophages (light blue) are seen in the top layer of the cortex. B cells (green) localize in the more superficial, while T cells (red) in the deeper areas of the LN. Most T and B cells exhibit a polarized shape and 'random' movement. Imaging depth: 87 μm; time elapsed: 9.5 min. Click here for file
