# Supplementary material for: Development of proteoglycan-induced arthritis depends on T cell-supported autoantibody production, but does not involve significant influx of T cells into the joints
Source: Arthritis Res Ther. 2010 Mar 18;12(2):R44. doi: 10.1186/ar2954 (PMC2888192; doi:10.1186/ar2954)
Supplement: Additional file 1 — Supplemental Figure 1 (Figure S1). Technical issues with in vivo joint imaging and rare cases of donor cell appearance in SCID joints after cell transfer. (a) Fluorescence-labeled cells injected directly into the joint cavity can be easily visualized by TPM. A mixture of CMTPX (red) and CMFDA (green)-labeled lymphocytes (~1,000 cells) was injected into the cavity of the ankle joint of a BALB/c mouse. The joint was subjected to TPM imaging 2 days after the intra-articular cell injection. Red and green fluorescent cells are visible in the joint in the vicinity of connective tissue (blue). (b) EGFP-expressing granulocytes (neutrophils) are abundant in the joints of EGFP-LysM KI mice with acute PGIA. Mice with EGFP knocked in the lysozyme M locus (EGFP-LysM KI), which express this fluorescent protein in their granulocytes at very high levels, were backcrossed into the BALB/c background and immunized to induce PGIA. TPM imaging was performed on the arthritic ankle joint 2 days after disease onset. Numerous green fluorescent cells are visible in the synovial tissue and cavity. (c) Fluorescence-labeled donor cells appear in the circulation of recipient SCID mice at early time points after transfer. Unseparated spleen-LN cells from arthritic donor mice were labeled with red CMTPX and injected i.v. into SCID recipients. TPM imaging of the ankle joint was carried out on the day following cell transfer. A single red fluorescent cell (arrow) is visible at a branching point of synovial blood vessels. Time-lapse video revealed a motility pattern that was similar to the behavior of neutrophils in EGFP-LysM KI mice, making uncertain that the red cell was a lymphocyte. (d-f) Donor cells are occasionally found in the synovial tissue or vessels after transfer of T cell-enriched fractions. (d) A red fluorescent cell (arrow) is visible in the synovial tissue 1 day after i.v. transfer of CMTPX-labeled T cells (along with CMFDA-labeled APCs) from arthritic donors to a SCID mouse. (e) High [file ar2954-S1.PDF]

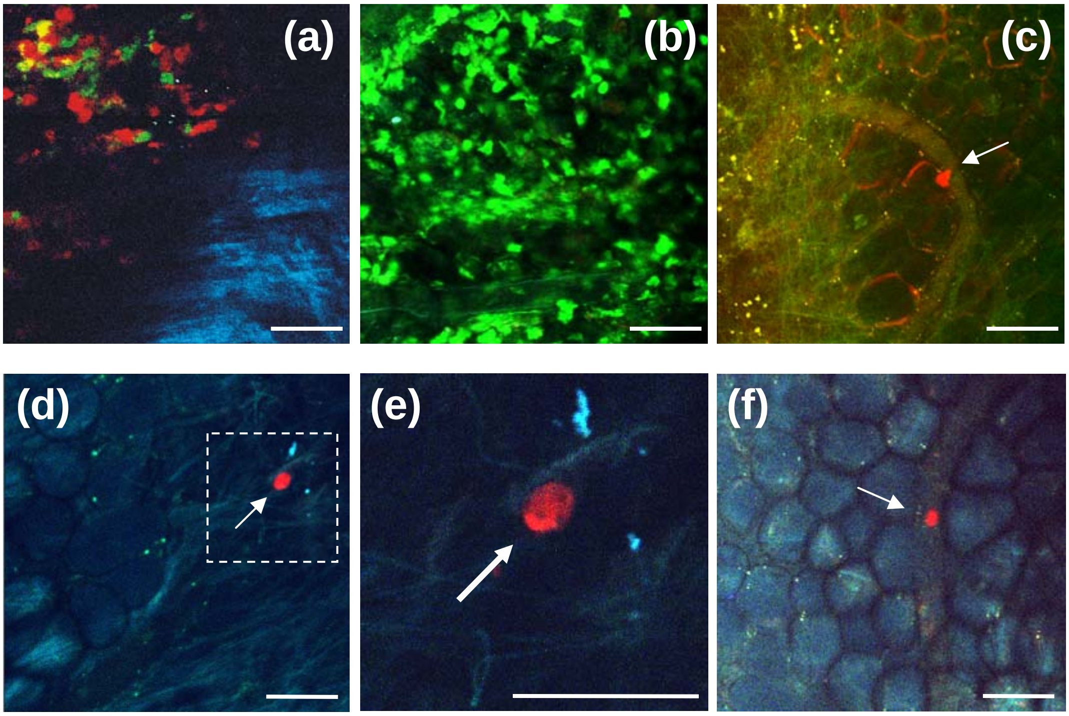

Figure S1. Technical issues with in vivo joint imaging and rare cases of donor cell appearance in SCID joints after cell transfer. **(a)** Fluorescence-labeled cells injected directly into the joint cavity can be easily visualized by TPM. A mixture of CMTPX (red) and CMFDA (green)-labeled lymphocytes (~1,000 cells) was injected into the cavity of the ankle joint of a BALB/c mouse. The joint was subjected to TPM imaging 2 days after the intra-articular cell injection. Red and green fluorescent cells are visible in the joint in the vicinity of connective tissue (blue). **(b)** EGFP-expressing granulocytes (neutrophils) are abundant in the joints of EGFP-LysM KI mice with acute PGIA. Mice with EGFP knocked in the lysozyme M locus (EGFP-LysM KI), which express this fluorescent protein in their granulocytes at very high levels, were backcrossed into the BALB/c background and immunized to induce PGIA. TPM imaging was performed on the arthritic ankle joint 2 days after disease onset. Numerous green fluorescent cells are visible in the synovial tissue and cavity. **(c)** Fluorescence-labeled donor cells appear in the circulation of recipient SCID mice at early time points after transfer. Unseparated spleen-LN cells from arthritic donor mice were labeled with red CMTPX and injected i.v. into SCID recipients. TPM imaging of the ankle joint was carried out on the day following cell transfer. A single red fluorescent cell (arrow) is visible at a branching point of synovial blood vessels. Time-lapse video revealed a motility pattern that was similar to the behavior of neutrophils in EGFP-LysM KI mice, making uncertain that the red cell was a lymphocyte. **(d-f)** Donor cells are occasionally found in the synovial tissue or vessels after transfer of T cell-enriched fractions. **(d)** A red fluorescent cell (arrow) is visible in the synovial tissue 1 day after i.v. transfer of CMTPX-labeled T cells (along with CMFDA-labeled APCs) from arthritic donors to a SCID mouse. **(e)** High-magnification imaging of the midsection of the same immobile red cell (boxed in panel d) revealed the presence of cytoplasmic “holes” corresponding to nuclear lobes. This raised the suspicion of this red cell being a polymorphonuclear leukocyte from the donor spleen cell preparation. **(f)** This SCID joint was imaged 4 days after the transfer of CMTPX-labeled T cells and unlabeled APC from arthritic donors. A single red cell (arrow) is seen within the shadow of a blood vessel. Serial images showed tethering-rolling movement of this red cell along the vessel walls, indicating that it was located intravascularly. The presence of donor cells in the recipients’ joints was not typical, and was even less frequent at later time points (days 7-18) after transfer. Scale bars, 50 μm.
